# Convergent Reduction of Ovariole Number Associated with Subterranean Life in Beetles

**DOI:** 10.1371/journal.pone.0131986

**Published:** 2015-07-07

**Authors:** Arnaud Faille, Dominique Pluot-Sigwalt

**Affiliations:** 1 Department of Entomology, Zoologische Staatssammlung, Munich, Germany; 2 Museum national d’Histoire naturelle, Département Systématique et Evolution, Institut de Systématique, Evolution, Biodiversité, UMR 7205 CNRS/MNHN, Paris, France; University of Maryland, UNITED STATES

## Abstract

**Background:**

Some species of obligate cavernicolous beetles are known to possess a unique feature—a contraction of the larval cycle. In contrast to many other subterranean beetles, life-cycle contraction in Trechini ground beetles (Carabidae) is correlated with a reduction in the number of eggs and a drastic reduction in the number of ovarioles. This remarkable peculiarity has only been reported for a small number of closely related species.

**Results:**

We give a description of the female internal reproductive system for six species of Trechini, including five subterranean species, with a particular focus on the western Pyrenean radiation of *Aphaenops*, a group for which nothing is known regarding the early life stages. We redescribe the internal female genitalia of *A*. *crypticola* Linder. Study of the ovarioles allowed us to infer the postembryonic development of the larvae for each species examined. We then used a phylogenetic framework to recognize two independent reductions in the number of ovarioles in the Pyrenean lineage. We discuss the multiple convergent evolutions in ovariole number and the potential link between a reduction of ovariole number and troglobiomorphism in a phylogenetic context.

**Conclusions:**

There is an extreme reduction in ovariole number and size within the species studied; the eggs produced by small ovarioles have a remarkably large size. A reduction to one ovariole has occurred independently at least twice in this subterranean group. A reduction in the number of ovarioles in ground beetles is one of the striking consequences of subterranean specialization and it is correlated with another remarkable adaptation of subterranean beetles, a reduction in the number of larval instars.

## Introduction

Variability in ovariole number in Insects has been the subject of numerous studies, particularly for *Drosophila* (see [1–2] for a review). The number of ovarioles per ovary may be influenced by inheritance, environmental differences or a particular way of life. For instance, temperature and food availability for the larva have a direct impact on variation in ovariole number, as observed in Coleoptera, Diptera and Hymenoptera [3–5]. The fecundity of animals with many ovarioles is more sensitive to nutrition than that of animals with fewer ovarioles [3]. Hodin suggested that plasticity in the number of ovarioles might be more prevalent for organisms living in fluctuating environments [1]. Therefore, stability in environmental conditions, as observed in subterranean ecosystems, may lead to stability in the number of ovarioles.

Organisms living in subterranean environments (also defined as hypogean species) tend to show a highly modified morphology and biology, combining adaptations resulting in the loss of some traits (e.g., eye degeneration, depigmentation) with enhancement of others, such as mechanical or chemical sensory organs and body shape modifications. These morphological adaptations are often associated with changes in the life cycle and metabolism [6–10]. Subterranean species tend to develop K-selected life history strategies, such as the production of fewer but much larger eggs, longer life expectancy and in some cases a reduction in the number and duration of larval instars [11].

Although hundreds of species of subterranean Coleoptera have been described to date, their biology is virtually unknown with regard to larval development. Jeannel hypothesized that the complete lack of larval data for Pyrenean subterranean Carabidae (Trechini) was the result of their special ecological requirements preventing their observation [12–13]. To explain the absence of larvae in caves where the adults are abundant, he suspected that the larvae were found in deep cracks of calcareous massifs, making them difficult to observe and study [13]. The only studies on the life cycles of subterranean Coleoptera were carried out by Deleurance, who bred representative species from the two most speciose groups of terrestrial subterranean beetles, namely Leptodirini (Leiodidae or “Bathysciinae”, Polyphaga) and the Trechini (Carabidae, Adephaga). She discovered that in the most highly specialized subterranean species of Leptodirini, the female lays only one enormous egg, out of which hatches a larva that does not feed and pupates directly without molting [14–15]. For other Leptodirini, the contraction of the life cycle is less radical, being intermediate between this extreme and a epigean life cycle. In such cases the larva still feeds, but the number of larval instars is less than in epigean species [15–16].

With regard to carabid subterranean beetles, Deleurance published some preliminary results [15, 17–20] suggesting that the same convergent evolution occurs in subterranean species as that observed for Leptodirini. By breeding two troglobitic species of the genus *Aphaenops*, she was able to obtain the first larval instar and observed that it does not feed. Unfortunately, because she was mainly studying Leiodidae, her results on Carabidae remain incomplete. Contrary to what she observed in Leptodirini, Deleurance noticed that the contracted life cycle of Trechini ground beetles is associated with a reduction in the number of ovarioles [19]. This observation suggests that the number of ovarioles could allow to predict the lack of active larvae in species whose ontogeny remains unknown.

Carabidae of the tribe Trechini that have diversified in karstic areas of the world have independently developed similar characteristics: they are completely blind and apterous, and they have a slender body form, sometimes showing an extreme elongation of head, pronotum and appendages, resulting in a very characteristic appearance—the ‘‘aphaenopsian” habitus [8, 21–23]. Whereas hypogean Trechini from the Alps comprise various biogeographic lineages [24], the Pyrenees harbors an endemic radiation of more than 80 species of hypogean Trechini belonging to the genera *Aphaenops* and *Geotrechus* [25]. The genus *Aphaenops* is not monophyletic in its current sense, and has two distinct radiations in the Pyrenees, the eastern and western clades sensu Faille et al. [25]. The only species for which the number of ovarioles is known [15] belong to the eastern clade (subgenus *Cerbaphaenops*), with nothing being known for the species of the western clade.

Here we describe the internal genital system of three representatives of the genus *Aphaenops* sensu stricto. *Aphaenops* (*Aphaenops*) is a monophyletic clade that has diversified in the western Pyrenees [25–26]. No information on the first instars was previously available [27]. We also studied the female internal system for a species in the subgenus *Aphaenops* (*Cerbaphaenops*), and two species belonging to two other genera, one endogean (*Geotrechus*) and one epigean (*Trechus*). All the species studied are Pyrenean microendemics except *Trechus bordei* Peyerimhoff, 1909, which is endemic to the western Pyrenees and Asturias [28].

## Materials and Methods

### Ethics statement

No specific permits were required for collecting the Trechini species of the genera *Trechus* and *Geotrechus* used for this study in France, because these genera are not protected. Licenses for collecting the four species of *Aphaenops* protected in France were obtained from the Directions Régionales de l’Environnement of the Midi-Pyrénées (*A*. *crypticola*, *A*. *leschenaulti*) and Aquitaine (*A*. *loubensi*, *A*. *ochsi*) (arrêtés N°2003–06, 25/2003, 2004–01). None of the species sampled are included in international lists for the conservation of endangered species of wild fauna (IUCN Red List of Threatened Species, Annexes of the Habitat Directive 92/43/EEC, Bern Convention or CITES).

### Species studied

One of the major challenges with subterranean beetles is to keep them alive from the cave to laboratory. Specimens were collected by hand, directly placed in a plastic container with humid moss and brought to the laboratory where they were anesthetized with ethyl acetate. We used four species of *Aphaenops* of the subgenera *Aphaenops* s. str. and *Cerbaphaenops*. We also studied one species of *Geotrechus* (*G*. *gallicus*) and one species of *Trechus* (*T*. *bordei*). We focused on the western clade of *Aphaenops*, with three representatives belonging to three distinct subclades: *A*. *leschenaulti* Bonvouloir, 1861, *A*. *loubensi* Jeannel, 1953, and *A*. *ochsi cabidochei* Coiffait, 1959 [25].

### Dissections

Anesthetized females were dissected in a saline solution (0.9%). After removal of the dorsal abdominal side and the intestine, the genital tract and the ovaries can be observed in place if they are not embedded in too much body fat. When the body fat was abundant, it was removed manually. Specimens were placed in a cavity slide and illustrated both before and after immersion in 70% ethanol to clarify some details inside the ovaries, such as the presence or absence of trophocytes and oocytes. All dissected parts were returned to alcohol for preservation.

Dry specimens were used for the examination of the ectodermal genital tract restricted to the cuticular intima, as many details are indiscernible on living specimens. Female abdomens were cleared in a 10% solution of potassium hydroxide (KOH) at room temperature for one to two days, then rinsed in water, placed in glycerol and dissected. The genital tract was then stained with Chlorazol Black E, placed in a cavity slide containing glycerol, and examined and drawn using a light microscope with a camera lucida. Pieces of specimens were stored in glycerol after examination. We follow the terminology of Deuve [29] for the female ectodermal genitalia.

### Phylogenetic analysis

The phylogeny was constructed using the following DNA fragments from selected species: two mitochondrial (5’ end of cytochrome c oxidase subunit 1 (*cox1*) and 5’ end of large ribosomal unit plus the Leucine transfer plus the 3’ end of NADH dehydrogenase subunit 1 (*rrnL*+*trnL*+*nad1*)) and two nuclear (small (SSU) and large (LSU) ribosomal units). Sequence data for all loci were generated in [24–25, 30] and available on GenBank (S1 Table). We aligned the sequences using the MAFFT online v.6 with the Q-INS-i algorithm and default parameters [31]. Maximum likelihood analyses were conducted on a combined data matrix with RAxML GUI [32–33], using three partitions corresponding to the *cox1*, *rrnL+trnL+nad1* and nuclear fragments, with a GTR+I+G evolutionary model. We used the default values for other parameters of the search [32]. Character states were mapped to the tips of the phylogeny.

## Results

Within the species of Trechini studied to date, the ovariole number is highly variable, from one to seven (see Table 1).

**Table 1:** Number of ovarioles per ovary within the subterranean beetle tribe Trechini (the number of individuals examined for the present study is indicated in brackets).

Genus	species	Habitat	Ovarioles number	Authors
***Trechus***	*noricus* Meixner	Ep	3–5	[34]
	*regularis* Putzeys	Ep	4–5	[34]
	*ovatus* Putzeys	Ep	5	[34]
	*pilisensis* Csiki (as *palpalis* Dej.)	Ep	7	[34]
	*bordei* Peyerimhoff	Ep	5–7	Present study (4)
***Duvalius***	sp.	H (End)	4	[19]
	*(Duvaliotes)* sp.	H (End)	3	[19]
***Trichaphaenops***	*gounellei* (Bedel)	H	3	[19]
***Typhlotrechus***	*bilimeki istrus* J. Müller as *dimnicensis* Müller)	H	6	[19]
***Geotrechus***	*orpheus* (Dieck)	H (End)	2	[19]
	*gallicus* (Delarouzée)	H (End)	2?	Present study (1)
***Aphaenops***	*ehlersi* (Abeille de Perrin)	H	2	[19]
	*cerberus* (Dieck)	H	1	[19]
	*crypticola* (Linder)	H	1	[19]
		H	1	[19]
	*hustachei* Jeannel	H	1	[19]
	*pluto* (Dieck)	H	1	[19]
	*tiresias* (Piochard de la Brûlerie)	H	1	[19]
	*loubensi* Jeannel	H	1	Present study (2)
	*ochsi cabidochei* Coiffait	H	1	Present study (2)
	*leschenaulti* Bonvouloir	H	1	Present study (1)

Habitats: End. = Endogean (living in the ground), Ep = Epigean (living on the ground), H = Hypogean (subterranean habitat).

We compared the ovaries and the female efferent genital ducts in different species. Most of the female specimens dissected were non-gravid and their ovarioles contained only a very young basal oocyte. A single female (*Aphaenops crypticola*) was gravid. We also observed the paired defense glands which are posteriorly located on each side of the genital efferent duct.

### Species having several ovarioles per ovary

**Fig 1:**
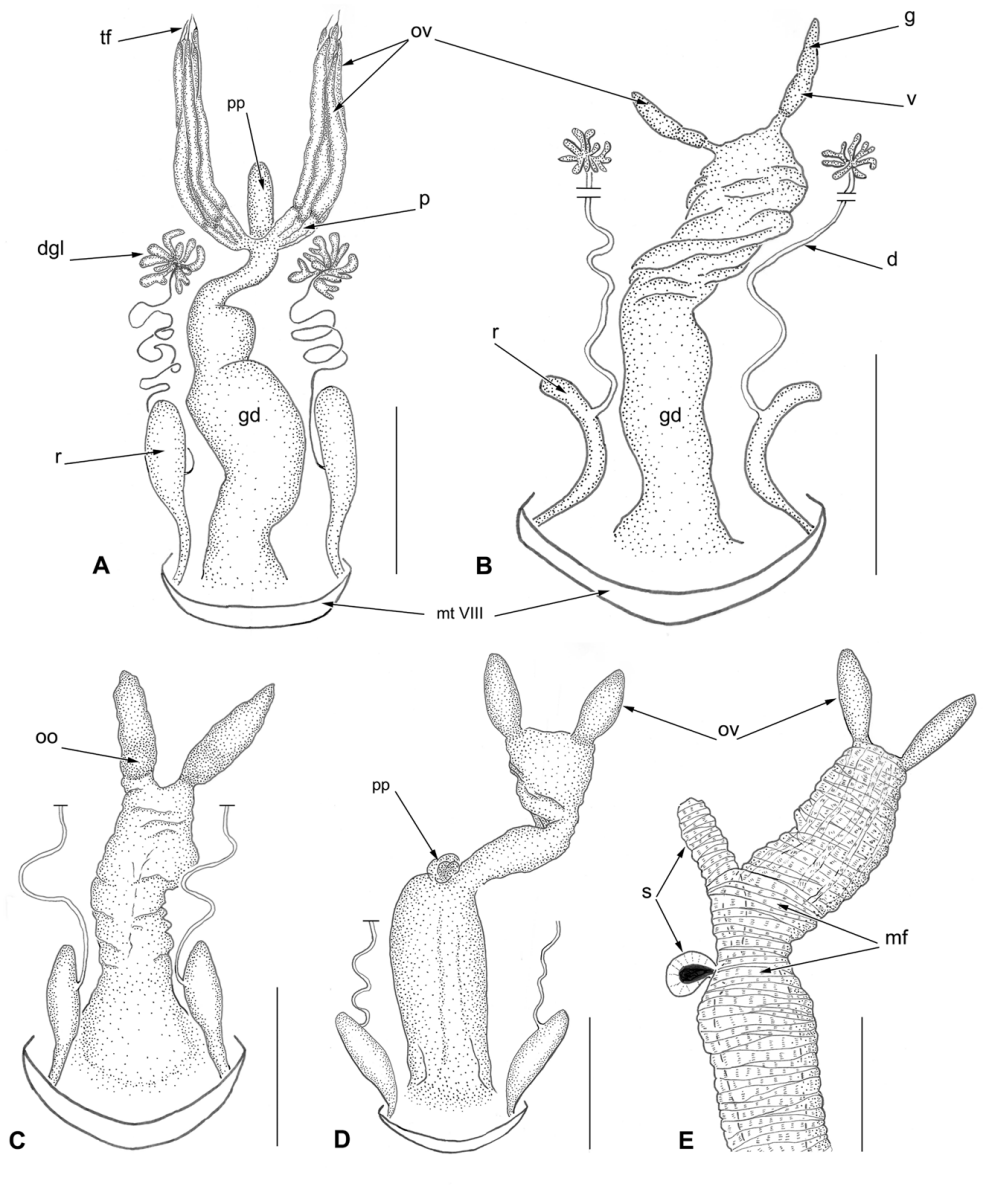
Female reproductive system and defense glands in five species of subterranean beetles of the tribe Trechini. Dorsal view, muscle fibers only shown on Fig 1E. (A) *Trechus bordei*. (B) *Aphaenops loubensi*. (C) *Aphaenops ochsi* ssp. *cabidochei* (apical acinous gland of defense glands not drawn). (D) *Aphaenops leschenaulti* (apical acinous gland of defense glands not drawn). (E) *Aphaenops crypticola* (paired defense glands not drawn). Scale bars: A = 5 mm; B, C, D = 1 mm; E = 0.5 mm. Abbreviations: cc = collecting canal of defense glands, dgl = defense gland, gd = genital duct surrounded by a muscular sheath, mf = muscle fibers, mt VIII = mediotergite VIII, oo = oocyte, ov = ovariole, pp = protruding part of genital duct, r = reservoir of defense glands, s = spermatheca, tf = terminal filament.

*Trechus bordei* possesses 5 to 7 polytrophic ovarioles per ovary (Fig 1A). The polytrophic type of ovariole is the rule within the entire group Adephaga. In polytrophic ovarioles, each follicle contains a number of trophocytes (nurse cells) together with one oocyte [35–37]. Ovarioles are tubular and elongate, attached together at the anterior end by their terminal filament. Germarium and vitellarium are distinct and the latter contains one or two small, immature basal oocytes. Pedicels are also elongated and seem to open directly into the median oviduct, the lateral oviducts being either extremely reduced or absent. The totality of the genital efferent system is enveloped in a thick layer of musculature (mainly circular muscle fibers, as illustrated in Fig 1E) to such an extent that the median oviduct, the vagina and the bursa copulatrix cannot be distinguished from each other. Between the ovaries there is a prominent median blind tube might be erroneously interpreted as a diverticulum of the genital efferent system, or a spermatheca or an accessory gland. However, treatment with KOH revealed that this tube is in fact the apical part of the bursa copulatrix tightly coated by a muscular sheath (see below). In one female specimen this tube-like projection was indistinct.

The ovariole number of *Geotrechus gallicus* was not clear and needs confirmation. Only a single female specimen was dissected and the ovaries were partly destroyed during the process. Nevertheless, in each ovary we were able to distinguish one well-developed ovariole plus several (2–3?) very thin ovariole-like structures in addition to the well-developed one. All the ovarioles were attached at the anterior tip by their terminal filament. The two well-developed ovarioles contained one or two small growing oocytes in the vitellarium. The thin ovariole-like structures appeared to be almost empty of cells. In *Geotrechus orpheus*, Deleurance-Glaçon [19] illustrated somewhat similar ovaries and counted two ovarioles per ovary.

Both species possess paired defense glands (pygidial glands) typical of Carabidae and the entire Adephaga [29, 38]. They open postero-laterally through the membrane between the mediotergites VIII-IX. The reservoirs are large; the acinous glands are deeply placed inside the abdomen and lie laterally on each side of the ovaries. They are connected to the reservoir by a remarkably long and convoluted collecting canal (Fig 1A).

### Species having only a single ovariole per ovary

**Fig 2:**
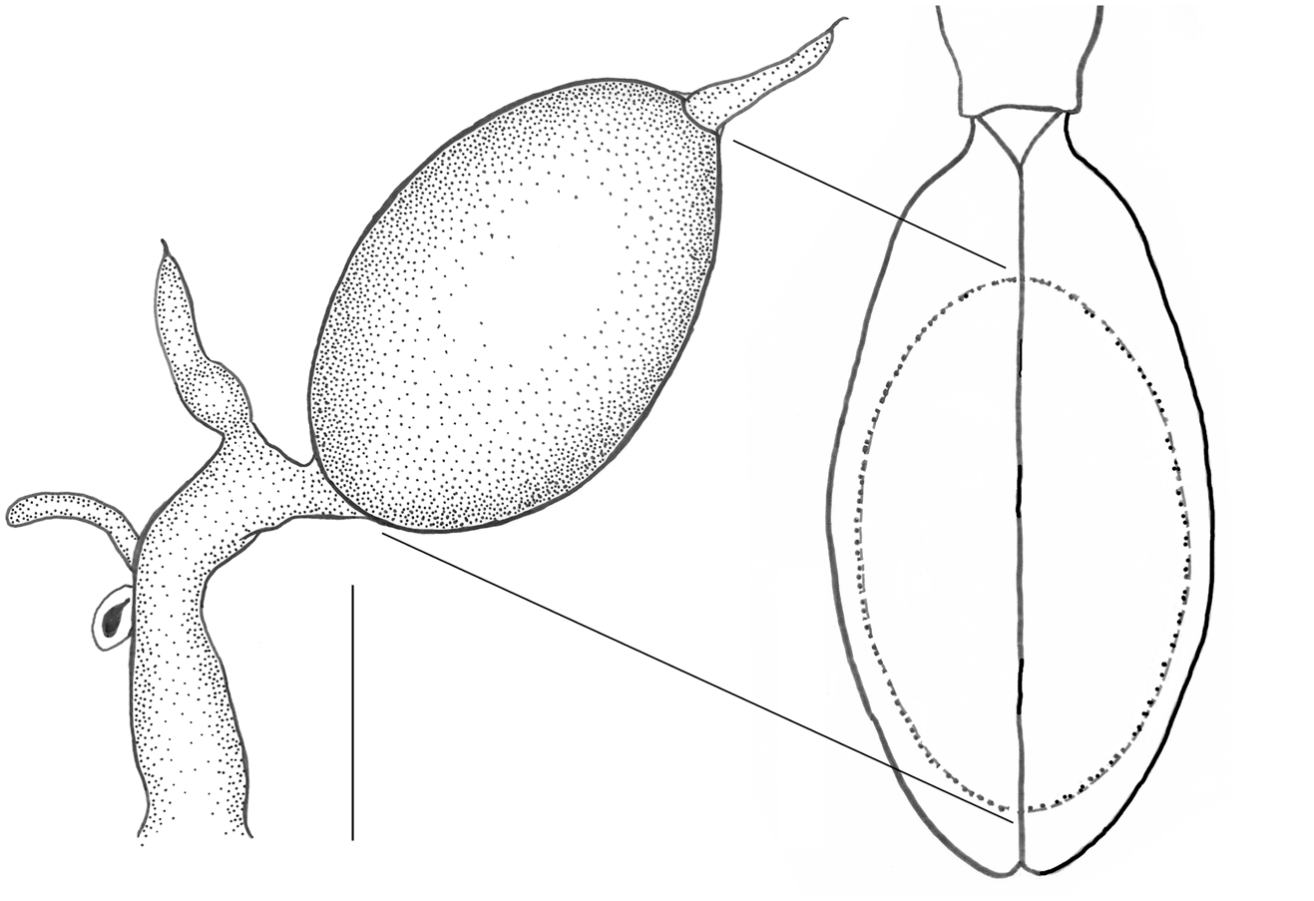
*Aphaenops crypticola*, oocyte. A gravid female with a mature oocyte in the right ovariole and the same oocyte in place within the female abdomen. Scale bar: 1 mm.

*Aphaenops loubensi* (Fig 1B), *A*. *ochsi* (Fig 1C), *A*. *leschenaulti* (Fig 1D) and *A*. *crypticola* (Fig 1E) possess only a single polytrophic ovariole per ovary. These single-ovariole ovaries are very small, somewhat sac-shaped, and usually embedded within a thick layer of fat body; the terminal filament is indistinct. The germarium and vitellarium are not distinct from each other, the pedicel is almost non-existent and lateral oviducts are probably absent. The other parts of the efferent genital duct—median oviduct, bursa copulatrix and vagina—are entirely enveloped in a dense, common sheath of musculature, such that they cannot be distinguished from one another. However, in *A*. *crypticola* (Fig 1E) a tube-like protrusion and a small pigmented vesicle project out of the thick muscle sheath; these structures were interpreted as forming the spermatheca (see below). One female of *A*. *crypticola* was gravid (Fig 2); the dextral ovariole contained a single, conspicuous, mature egg, with numerous, large, lipid-like spherules. As shown in Fig 2, the 2.1 mm long egg occupied most of the abdominal cavity, representing nearly half the length of the female (4.5mm). In contrast, the left ovariole appeared minute, containing only a small basal oocyte. The two ovarioles may function alternately.

The paired defense glands are present in the four *Aphaenops* species examined. As in *Trechus bordei*, the acinous glands are located at the level of the ovariole and the reservoirs do not show any sign of reduction.

### The female ectodermal genital tract

**Fig 3:**
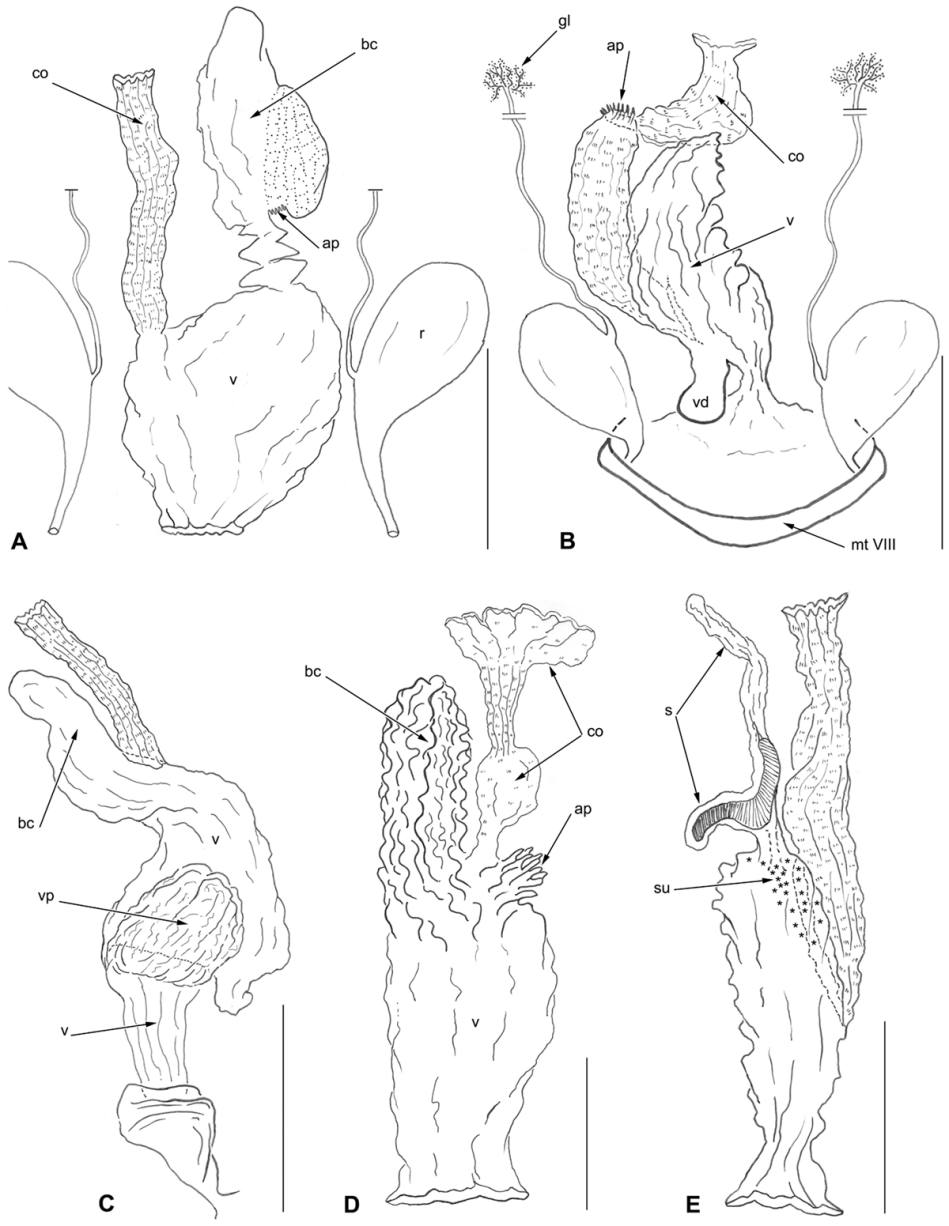
Female ectodermal genital ducts restricted to their cuticular intima lining by KOH treatment. Dorsal and more or less lateral views in order to show the different elements. (A) *Trechus bordei*. (B) *Aphaenops loubensi*. (C) *Aphaenops ochsi* ssp. *cabidochei*. (D) *Aphaenops leschenaulti*. (E) *Aphaenops crypticola*; the vaginal secretory units are indicated by asterisks. Scale bars: A = 5 mm; B = 1 mm; C-E = 0.5 mm. Abbreviations: ap = apodeme-like structure, bc = bursa copulatrix, co = common oviduct, gl = defense gland, mt VIII = mediotergite VIII, pp = protruding part of genital duct, s = spermatheca, su = secretory unit, v = vagina, vd = vaginal diverticulum, vp = vaginal pouch.

Examination of the female ectodermal genital tract after KOH treatment revealed several details through the cuticular intima lining. The female genital ducts of the five species examined are illustrated in Fig 3. A full description falls out of the scope of this paper and we only treat their general characteristics here, which have already been observed by Deuve [29] in other Trechini. There is a great diversity in the architecture of the genital ducts, even within the same genus (Fig 3B–3E). The following features are noticeable: i) the common oviduct, located ventrally as in other Adephaga, which is either long and straight or curiously collapsed upon itself (Fig 3B), as it is the rule in many insects, its intima bears internal minute spines; ii) the bursa copulatrix is present (Fig 3A, 3C and 3D) or absent (Fig 3B and 3E); iii), the spermatheca is lost, except in *A*. *crypticola* (Fig 3E); iv), the vagina is wide, being either simple (Fig 3A, 3D and 3E) or equipped with a posteriorly rounded diverticulum (Fig 3B) or with a large dorsal pouch (Fig 3C). Furthermore, in *A*. *crypticola* alone, we have observed numerous cuticular ductules scattered on the vaginal intima close to the opening of the spermatheca (Fig 3E), these being the efferent ductules of the secretory units of class III according to the classification of Noirot and Quennedey [39]. Except for these scattered secretory units, no glands are associated with the genital ducts.

## Discussion

### Variation in ovariole number in beetles

In Coleoptera, the number of ovarioles per ovary can vary from one to 200 [40], and even up to 1000 in the genus *Meloe* (Meloidae) [37]. As stated by Robertson [40], during the diversification of Coleoptera the ovariole number has either been multiplied or reduced within groups, whereas in others, such as in Curculionidae, it has remained highly constant. In Carabidae, as for all Adephaga, ovariole number is still poorly documented, but it appears to be highly variable. Each ovary may contain from 4 to 40 ovarioles in Adephaga and from 4 to 18 in Carabidae [40]. In the Trechinae, although only known for a few species, the number of ovarioles ranges from 1 to 7 [19] (see Table 1).

### Reduction of ovariole number and specialization of life history strategy

In several groups, it is clear that the reduction of the ovariole number has been influenced by the life history of the species. For example, a reduction in the number of ovarioles is correlated with nesting behavior in Hymenoptera [41] and Scarabaeoidea [42]. The lowest number of ovarioles in Coleoptera is 1 or 0 in dung beetles (Scarabeidae s. str.) and this single ovariole is the left unpaired ovary typical of the family [43–47]. In this group, the extreme reduction of the ovariole number (1–0) is clearly related to the complex nesting behavior, with the digging of a nest and provisioning for each egg, so that the larva has all the food required for its development [42,46]. The great investment of the female—with the cooperation of the male in several species—does not allow for high fecundity and thus reduces the number of eggs laid. The loss of an ovary is exceptional in insects. Within Coleoptera it has been reported in *Bledius* spp. (Staphylinidae), a subsocial genus living in intertidal areas [48]. In this genus, females exhibit parental care [48–50]. However, in this case the remaining ovary has 6 ovarioles, the typical number for the family. Drastic ovarian regression to 1–0 ovarioles, as in dung beetles, is also observed in the holocyclic aphid species with sexual generation, particularly in the Phylloxeridae and Adelgidae, in which female sexuales, after mating, deposit a single egg (the “overwintering egg”) and then die (see [51–54]). Moreover, within a given aphid species, the number of ovarioles is highly variable between generations [55].

In social insects (Hymenoptera) ovariole reduction occurs in the workers, which possess very few ovarioles compared to the queen. In *Cremastogaster* (Formicidae), each ovary is reduced to one ovariole [56]. In *Apis mellifera* ovarioles vary from 1 to 30 [57–58].

Except for *Drosophila* (see [2]), very little is known about the ontogenetic process underlying ovariole differentiation. As stated by Green [2], the larval stages represent a critical period during which ovariole number is determined. In Scarabaeidae (Coleoptera), the reduction in ovariole number takes place during postembryonic development, after the formation of two symmetric gonads in which six ovarioles are differentiated, as in most Scarabaeoidea [59]. All the ovarioles, save one in the left ovary, are apparently sterile, remaining atrophied during organogenesis and finally disappear [59]. In several aberrant cases (females belonging to *Scarabaeus*, *Onthophagus* and *Canthon*) the persistence of a second either functional or non-functional ovariole in the left ovary has been observed [44, 59–61]. These cases show that the sequence of events may have broken down during organogenesis.

### Reduction in ovariole number and subterranean evolution

Various factors affecting the natural history of hypogean animals (scarcity of food in subterranean ecosystems, stable conditions and flightlessness) lead to a reduction in ovariole numbers. This may reflect the evolution of an extreme K-strategy in a harsh and stable environment [11]. The adaptation of animals to a subterranean life leads to remarkable morphological and physiological convergences [6,8,10,62–63]. In *Aphaenops*, it is likely that the ovarian regression is genetically determined and not environmental. This ovarian reduction is strongly correlated with both the very large eggs produced and the contraction in number of larval stages. *Aphaenops* lay eggs that are remarkably large in comparison with the body size of the female (e.g., ca. half of the body length of the female in *A*. *crypticola*, Fig 2). The reduction in ovariole number is very often associated with the large size of mature eggs in insects (e.g., [41,56,64]). The most striking example is the single “overwintering egg” laid by the female aphids during the sexual generation of Phylloxeridae and Adelgidae. In the woolly apple aphid (*Eriosoma lanigerum* Hausmann), the mature egg occupies the whole of the abdominal and thoracic cavity (see Figure 254 in [52]). In *Aphaenops crypticola*, the mature egg fills almost all the abdominal cavity. The huge eggs of *Aphaenops* spp. might be related to the contraction of the larval phase and might supply the non-feeding larva with the elements needed to complete its development. These elements are the proteid yolk (vitellins), which represents the major component of insect eggs, along with lipids and carbohydrates, which are present in considerable amounts [64]. Food supply is known to have a direct impact on the number of eggs laid in Carabidae [65]. The scarcity of food in caves may also impact the strategy of a reduction in the number of eggs laid. In beetles of the family Silphidae, flightlessness and food habit change are also known to be associated with a decrease in ovariole number and an increase in egg size [66]. The correlation between a reduction in ovariole number and subterranean specialization was first noted in Canarian cockroaches of the genus *Loboptera* (Dictyoptera, Blatellidae) [67]. Within Coleoptera, Deleurance-Glaçon noticed that although the correlation between troglobiomorphism, a shortening of the larval phase and a reduction in ovariole number is apparent within Trechini, this is not the case for Leptodirini (Leiodidae). In the latter group, the specialization to subterranean life is associated with a reduction in the number of larval instars, but this adaptation appears not to be correlated with a reduction of the ovariole number [19].

### Implications regarding the evolution of Trechini

**Fig 4:**
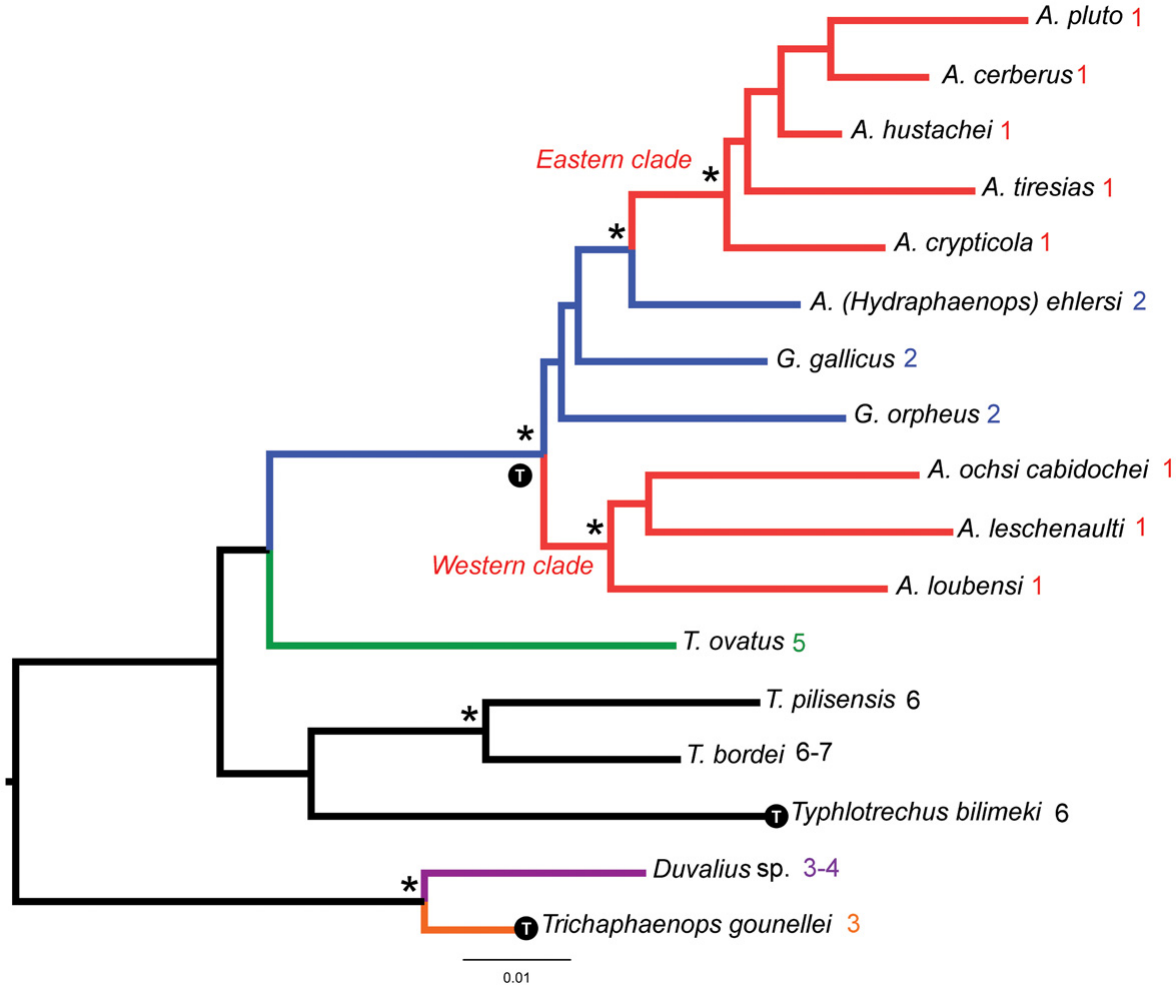
Number of ovarioles mapped on simplified phylogram of Trechini. Simplified phylogram of Trechini obtained with RAxML using a combined data matrix of mitochondrial (*cox1*, *rrnL*+*trnL* and *nad1*) and nuclear (LSU, SSU) markers. Troglobitic species marked with a “T” in a black circle. *: bootstrap >95. The number of ovarioles per ovary is indicated after the species name. Colors indicate number of ovarioles: red: 1, blue: 2, orange: 3, violet: 3–4, green: 5, black: >5.

All the species of *Aphaenops* studied to date share the state of an ovary with a single ovariole. The genus *Aphaenops* is not monophyletic, and is represented by two independent subterranean radiations in the Pyrenees, one in the Eastern part (*Cerbaphaenops*), one in the Western part of the range (*Aphaenops* s. str.) [25]. *Hydraphaenops*, which is not monophyletic, was recently considered as a subgenus of *Aphaenops*, together with a new subgenus, *Simaphaenops* [26]. Most of the species belonging to these two groups are known by very few specimens, and the only species for which the number of ovarioles is known is *A*. (*Hydraphaenops*) *ehlersi* [15,19]. It has two ovarioles per ovary and an active larva ([19]; Table 1). Because *A*. (*H*.) *ehlersi* is phylogenetically closer to *Cerbaphaenops* than to the western *Aphaenops* s. str., the presence of more than one ovariole per ovary in this species indicates an independent reduction within the Pyrenean lineage of subterranean Trechini (Fig 4). The reduction to one ovariole has therefore occurred at least twice in *Aphaenops*. Other evidence that such a reduction occurred twice in the Pyrenean lineage is the presence of at least two ovarioles in the species of *Geotrechus* studied. Because the genus *Geotrechus* is not monophyletic [25], the regression in ovariole number in the two cave-adapted lineages of Trechini (eastern and western radiations) suggests an independent regressive evolution in the Pyrenean lineage. Unlike the Pyrenean species, the number of ovarioles is high in the Adriatic subterranean species *Typhlotrechus bilimeki* Sturm ([19]; Table 1). *Typhlotrechus* originated in the Oligocene and underwent little diversification (two species only) [21,24].

Many species of *Duvalius* are troglobitic and have undergone a reduction in the number of ovarioles, as in the subterranean species of the genus *Trichaphaenops*. This reduction is less drastic than in *Aphaenops*. Although troglobitic, they still possesses two ovarioles per ovary, as in non-troglomorphic representatives of *Duvalius*, but we do not know whether the species of *Duvalius* mentioned by Deleurance (Table 1) is a subterranean species or not, since it was not identified to species [19]. The *Duvalius* lineage diversified much more recently than the Pyrenean lineage, and the origin of the hypogean genus *Trichaphaenops* dates from the Plio- or Pleistocene, whereas the Pyrenean lineage originated in the Early Miocene [24]. This suggests a possible link between the age of the subterranean lineage and the degree of reduction in the number of ovarioles. The number of ovarioles within the genus *Trechus* varies from 3 to 7 according to the few species studied so far, but further investigations are required since this genus is very speciose, widespread and polyphyletic, with species colonizing various habitats, from high altitude mountains to subterranean environments [24, 68–69].

## Conclusions

The present study confirms and extends the preliminary results obtained by Deleurance [14–15, 17–18, 19–20]. In this study we have demonstrated that: (1) there is an extreme reduction in ovariole number within *Aphaenops*, the number of ovarioles per ovary being 1–1 (as opposed to 2 to 6 in other Trechini subterranean beetles); (2) this single ovariole is strongly reduced in size compared to the elongated ovarioles observed in those subterranean beetle species that have several ovarioles per ovary; (3) the eggs produced by this small ovariole have a remarkably large size. This ovarian reduction and the great size of the eggs seem to be correlated with a subterranean life and the contraction of larval phases observed by Deleurance [15] (see below).

By contrast, the reduction to a single ovariole per ovary is not accompanied by modification of the genital ducts. In *Aphaenops* spp., the ectodermal genital tract is similar to that described by Deuve [29] in various species of Trechini, especially *Trechiama siamensis* Deuve. From our data and the large comparative study by Deuve [29] on Adephaga as a whole, subterranean life might be correlated to regression and modification of parts of the genital ducts, i.e., loss of the spermatheca and its gland, loss of the bursa copulatrix, or bursa oddly or weakly differentiated, vagina dilated, and absence of accessory glands. The defense glands of *Aphaenops* do not show any unusual modifications. The reduction in the number of ovarioles in subterranean ground beetles is one of the striking consequences of subterranean specialization, and is apparently correlated with another remarkable adaptation of subterranean beetles, the reduction in the number of larval instars. In Leiodidae Leptodirini, the other main Palearctic subterranean radiation of beetles, this contraction of the larval phase is correlated with an increase in diversification rate [16].

## Supporting Information

S1 Table Material used in the study, with locality data, voucher number and accession numbers of the sequences. (XLS)
